# Molecular Mechanisms of Microcystin Toxicity in Animal Cells

**DOI:** 10.3390/ijms11010268

**Published:** 2010-01-21

**Authors:** Alexandre Campos, Vitor Vasconcelos

**Affiliations:** 1 Centro Interdisciplinar de Investigação Marinha e Ambiental, CIIMAR/CIMAR, Rua dos Bragas 289, 4050-123 Porto, Portugal; E-Mail: vmvascon@fc.up.pt; 2 Departamento de Biologia, Faculdade de Ciências da Universidade do Porto, Rua do Campo Alegre, 4069-007 Porto, Portugal

**Keywords:** cyanobacteria, microcystins, animal cells, mitochondria, apoptosis

## Abstract

Microcystins (MC) are potent hepatotoxins produced by the cyanobacteria of the genera *Planktothrix*, *Microcystis*, *Aphanizomenon*, *Nostoc* and *Anabaena.* These cyclic heptapeptides have strong affinity to serine/threonine protein phosphatases (PPs) thereby acting as an inhibitor of this group of enzymes. Through this interaction a cascade of events responsible for the MC cytotoxic and genotoxic effects in animal cells may take place. Moreover MC induces oxidative stress in animal cells and together with the inhibition of PPs, this pathway is considered to be one of the main mechanisms of MC toxicity. In recent years new insights on the key enzymes involved in the signal-transduction and toxicity have been reported demonstrating the complexity of the interaction of these toxins with animal cells. Key proteins involved in MC up-take, biotransformation and excretion have been identified, demonstrating the ability of aquatic animals to metabolize and excrete the toxin. MC have shown to interact with the mitochondria. The consequences are the dysfunction of the organelle, induction of reactive oxygen species (ROS) and cell apoptosis. MC activity leads to the differential expression/activity of transcriptional factors and protein kinases involved in the pathways of cellular differentiation, proliferation and tumor promotion activity. This activity may result from the direct inhibition of the protein phosphatases PP1 and PP2A. This review aims to summarize the increasing data regarding the molecular mechanisms of MC toxicity in animal systems, reporting for direct MC interacting proteins and key enzymes in the process of toxicity biotransformation/excretion of these cyclic peptides.

## 1. Introduction

Cyanobacteria are photoautotrophic bacteria commonly found in fresh and brackish waters all over the globe. The cyanobacteria in eutrophic waters and under specific environmental conditions can grow excessively, producing blooms. Cyanobacteria growth is a natural phenomenon, but it is becoming more frequent and severe as a result of anthropogenic activity. One particularity of cyanobacteria is the production of secondary metabolites which are toxic to other organisms. These toxins under situations of excessive cyanobacterial growth can be accumulated in aquatic wildlife and transferred to higher trophic levels with the risk of livestock and human poisoning. Therefore cyanobacterial blooms are regarded as a serious human health issue [1], also having a strong impact on wildlife [2,3].

**Figure 1.**
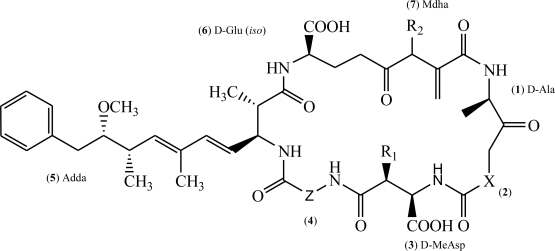
General structure of microcystin, adapted from McElhiney and Lawton [5]. In MC-LR X represents l-Leucine; Z l-Arginine; R_1_ and R_2_ CH_3_.

The microcystins (MC), produced by species of the genera *Planktothrix*, *Microcystis*, *Aphanizomenon*, *Nostoc* and *Anabaena* [4], are among the most frequently detected in fresh waters, and produce potent toxic effects. The general structure of microcystin (MC) is cyclo-(d-Ala1-X2-d-MeAsp3-Z4-Adda5-d-Glu6-Mdha7), where X and Z are variable l-amino acids, d-MeAsp represents d-erythro-β-methylaspartic acid, Adda the unusual amino acid (2*S*,3*S*,8*S*,9*S*)-3-amino-9-methoxy-2,6,8-trimethyl-10-phenyldeca-4,6-dienoic acid and Mdha *N*-methyldehydroalanine [4] (Figure 1).

Nevertheless there are identified over 80 variants of this molecule [6] including amino acid variations and modifications. The molecular weight of MC varies between 900 and 1,100 Daltons. Amino acid variations in positions X and Y account for many of the MC variants. The most common are MC-LR, MC-RR and MC-YR, with different combinations of leucine (L), arginine (R) or tyrosine (Y) [7]. MC-LR is the most studied due to its ubiquity, abundance and toxicity [8]. The Adda moiety, present in all variants, is critical to MC activity [9]. Its isomerization and/or oxidation dramatically reduces the toxicity [10]. Microcystins inhibit serine/threonine-specific protein phosphatases (PPs) such as PP1 and PP2A through the binding to these enzymes [11,12]. The acute toxicity of MC can be explained by this phosphatase inhibition which leads to an excessive phosphorylation of proteins and to alterations in cytoskeleton, loss of cell shape with subsequent destruction of liver cells causing intra-hepatic haemorrhage or hepatic insufficiency [4]. MC are also responsible for the increase of oxidative stress in cells which subsequently can trigger apoptotic processes [8,13]. These molecules have been regarded also as tumor promoters [14–18].

Human health problems are most likely related with chronic exposure to low MC concentrations through consumption of contaminated waters and food (agricultural products, fish, prawns, and mollusks), dermal exposure and inhalation. Liver is the most affected organ in humans but the exposure to the toxin is likely to affect organs such as kidney and colon as evidenced by *in vivo* [19–22] and *in vitro* studies [23,24]. Therefore the illnesses attributed to MC intoxication are gastroenteritis and related diseases, allergic and irritation reactions, and liver diseases. Some lesions can evolve into tumors and primary liver cancer and colorectal cancers in human populations have been related with MC exposure and toxicity [25–27]. It is important to bear in mind that MC effects are time and concentration dependent [28].

The MC are non-ribosomal peptides synthesized by a multi-functional protein complex containing non-ribosomal peptide synthetase (NRPS) and polyketide synthase (PKS) domains [29]. This multi-functional protein complex is present only in prokaryotes and lower eurakyotes [29] and is encoded by large bacterial gene clusters which comprises at least two operons, the *mcyABC* (peptide synthetase) and *mcyDE* (hybrid polyketide-peptide synthetase) [30]. Although the toxin can be localized in all regions in the interior of cyanobacteria cells, it was suggested that most of cell’s toxin complement is localized in the thylakoids, followed by the nucleoplasm and pyrophosphate bodies [31]. The association of MC to the thylakoids might occur through the hydrophobic ADDA moiety inserted in the membrane and the polar cyclic structure interacting with cytoplasm. This preferential localization suggests that the molecule may play an important role in light harvesting and adaptation responses [29]. MC can reach intracellular concentration far above the limit of solubility in water. For this reason it was recently suggested that this high concentration in M*icrocystis* cells can be reached by MC binding to phycobilins [32].

The molecular mechanisms of microcystins toxicity are being investigated and advances have been reached in the knowledge of the activity of this cyclic peptide at cellular and molecular level. Moreover the molecular basis of MC biotransformation is being elucidated. This review aims to analyze the information published so far regarding the molecular aspects of MC toxicity and bio-transformation in animal systems. A summary of the main proteins reported and their roles in MC mediated cell injury is presented in the Table 1.

## 2. Cellular Uptake of MC

MCs cannot readily diffuse through plasma membrane due to the high molecular weight and structure. It is also known that due to the cell specificity and organotropism of MC-LR, selective pathways of MC up-take might exist. Ericksson *et al*. [33] studied the cellular up-take of a radiolabelled derivative of MC-LR in isolated rat hepatocytes, human hepatocarcinoma cell line Hep G2 and the mouse fibroblast cell line NIH-3T3. The up-take was shown to be specific of hepatocytes. Moreover by means of a surface barostat technique the authors showed that the membrane penetrating capacity (surface activity) of MC-LR is low, indicating that the toxin requires an active uptake mechanism. The inhibition of MC up-take by the use of inhibitors of the bile acid transporters further suggested the requirement of this system for MC to enter the cells [33]. In fact many transporters are responsible for the uptake of endogenous and exogenous chemicals into liver, including the organic anion transporting polypeptides (Oatps), Nat-taurocholatecotransporting polypeptide (Ntcp), organic anion transporters (Oats), and organic cation transporters [34]. Among these transporters, the Oatps are thought to be responsible for the majority of hepatic uptake of chemicals, whereas Ntcp mainly transports bile acids into liver [35]. Recently Fischer *et al*. [36], using the *Xenopus laevis* oocyte expression system, studied the transport of MC-LR by different members of the organic anion transporting polypeptide superfamily (rodent: Oatps; human: OATPs). The authors verified that rat Oatp1b2, human OATP1B1, human OATP1B3, and human OATP1A2 transport MC-LR 2- to 5-fold above water-injected control oocytes. MC-LR transport was inhibited by co-incubation with the known Oatp/OATP substrates taurocholate (TC) and bromosulfophthalein (BSP). No MC-LR transport was observed in oocytes expressing other Oatp members (Oatp1a1, Oatp1a4, and OATP2B1), demonstrating in this way the MC specificity for rat Oatp1b2, human OATP1B1, human OATP1B3, and human OATP1A2. The OATP is a multispecific transport system expressed in diverse cell types such as enterocytes, hepatocytes and renal epithelial cells [6] and organs such as the heart, lung, spleen, pancreas, brain and blood-brain-barrier (BBB) [6]. Systemic distribution of MC in the organs will be therefore dependent on the degree of blood perfusion and types and expression level of OATP carriers. OATP1B1 and 1B3 are human liver specific while OATP1A2 is highly expressed in endothelial cells of the BBB, epithelial cells of the blood-cerebrospinal-fluid-barrier (BCFB) and in the cell membrane of human neurons [6]. Homologous to human OATP genes have been found in most diverse animals, some being related with the transport of xenobiotics including MC [37]. The importance of OATP system in MC toxicity was evidenced in Oatp1b2-null mice engineered by homologous recombination. Three hours after administration of microcystin-LR the binding of the cyclic peptide to the hepatic protein phosphatase 1/2a was much lower in Oatp1b2-null mice compared with wild-type mice. Livers of wild-type mice presented extensive hemorrhagic necrosis and elevated serum levels of both ALT and ALP also mortality was registered. Nevertheless Oatp1b2-null mice demonstrated complete resistance to hepatotoxicity induced by MC-LR [38].

## 3. Toxicity Mechanisms

### 3.1. Interaction with Protein Phosphatases PP1 and PP2A

**Figure 2.**
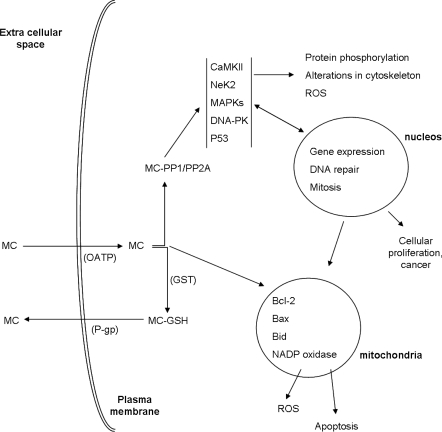
Suggested pathways of MC up-take, toxicity, biotransformation and excretion in animal cells.

One of the most studied pathways of MC activity is the inhibition of serine/threonine protein phosphatases by interacting with the catalytic subunits of these enzymes [39]. Protein phosphorylation/dephosphorylation is a dynamic process and an important pathway for the regulation of protein activity in cells. It is catalyzed by phosphatases and kinases. Therefore uncontrolled inhibition of these enzymes can have significant impact in cell’s homeostasis. MC have a strong affinity to PP1 and PP2A types but have little effects on PP2B [40,41]. The interaction of MC-LR with PP1 and PP2A was reported as a two step mechanism, where the toxin first binds to the enzyme inactivating it and subsequently forms covalent adducts during prolonged reaction-time [11,42]. The determination of the crystal structure of MC-LR binding to PP1 and PP2A was important to understand the nature of the interactions and the enzyme inhibition. MC-LR binds to the PP-1c α isoform through interactions at three sites of the enzyme, the hydrophobic groove, C-terminal groove and the catalytic site [43]. MC-LR coordinates with the two catalytic metal atoms of the phosphatase indirectly by binding two water molecules through the α-carboxyl group of its γ-linked d-glutamic acid moiety and the adjacent carbonyl oxygen. Therefore the glutamic acid α-carboxyl group plays a significant role in the toxicity of the cyclic peptide. The carboxyl group of the MeAsp residue of MC-LR interacts with Arg96 and Tyr134 of PP-1c blocking the access to the active centre of the enzyme [44]. Moreover the long hydrophobic tail, composed of the Adda residue interacts with the hydrophobic groove region of PP-1c, adjacent to the active site. Interactions can also occur at the toxin-sensitive b12–b13 loop (residues 268–281) of PP-1c. The crystal structure showed the covalent linkage between the Mdha side-chain of the toxin and the Cys-273 of PP-1c confirming the previous biochemical analyses. The crystal structure of MC-LR interacting with PP2A was described by Xing *et al*. [45]. The authors reported the binding of the toxin to the surface pocket of PP2A located above the two manganese atoms and the active site of the enzyme. The binding is strengthened by hydrophobic interactions between the Adda side chain of MC-LR and the residues Gln122, Ile123, His191, and Trp200 of the binding pocket, by van der Waals interactions with the hydrophobic portion of the toxin and the residues Leu243, Tyr265, Cys266, Arg268, and Cys269 of the binding pocket and, by a covalent linkage between the Sγ atom of Cys269 and the terminal carbon atom of the Mdha side chain [45]. The pathways involved in MC toxicity can be suggested by knowing the functions of PP1 and PP2A and their protein substrates. PP2A has been regarded as an important tumor suppressor. Much of this evidence results from the observation that the enzyme is inactivated by the potent tumor inducer okadaic acid and that mutations, modifications, gain or loss of activity, in the distinct subunits of the enzyme have been linked to the development of cancer cells [45]. Moreover the well known effect of MC on liver tissue organization, hepatocyte morphology and cytoskeleton structure/dynamics has been related with PP1/PP2A activity and increase in phosphorylation of cytoskeleton proteins [46–48], pointing out the role of these enzymes in the MC mediated acute toxicity and the importance of the identification of PP substrates for a comprehensive characterization of the pathways of MC action. In the next section of this review we will report the alterations in the activity/expression of phosphoproteins linked to MC mediated PP inhibition. The pathways of toxicity related with PP inhibition are represented in Figure 2.

### 3.2. Regulation of Activity/Expression of Phosphoproteins

Two pathways of DNA repair, the nucleotide excision repair (NER) and the DNA double strand break (DSB) repair by the nonhomologous end joining (NHEJ) have been shown to be inhibited by MC, pointing out the genotoxic attributes of the toxin [49,50]. Both pathways are regulated by phosphorylation and *in vitro* experiments demonstrated that PP1 and PP2A inhibition by significantly decreases the activity of the DNA repair systems [49,51]. Moreover the inhibition of the DSB-NHEJ is due to the phosphorylation-induced loss of the protein kinase activity of the DNA-dependent protein kinase (DNA-PK), through the direct inhibition of PP2A like enzymes [49]. The DNA-PK is a complex composed of a catalytic subunit (DNA-PKcs) and the DNA end-binding Ku70/Ku80 heterodimer. All three subunits undergo phosphorylation *in vitro* and *in vivo* [49].

The calcium-calmodulin-dependent multifunctional protein kinase II (CaMKII) is activated by Ca^2+^/calmodulin, autophosphorylation and limited proteolysis. CaMKII can be activated by caspases thereby contributing to the downstream events in apoptosis. Fladmark *et al*. [52,53] first reported that PP-inhibitor-induced protein phosphorylation in hepatocytes and apoptosis require active CaMKII. Ding *et al*. [54,55] postulated that the enzyme plays a role in the late events of MC induced cell death, being activated through the increase of intracellular Ca^2+^. In an attempt to elucidate whether this enzyme acts upstream or downstream of the commitment point of cell death, Krakstad *et al*. [56] screened cells for later morphological and functional signs of cell death, after adding cell-penetrating CaMKII inhibitors to microcystin-treated hepatocytes in pre-apoptotic stages. The authors verified that CaMKII-dependent commitment point precedes a putative reactive oxygen species (ROS) commitment point, suggesting that CaMKII activation occur upstream ROS formation. CaMKII activation is achieved by inhibition of its dephosphorylation. This might be under control of MC through the inhibition of PP1 and PP2A (Figure 2). CaMKII activation might further regulate downstream events such as ROS formation and phosphorylation of proteins including myosin light chain (MLC) [56].

The Nek2, a member of the NIMA-related serine/threonine kinase family, is a cell cycle dependent protein kinase that participates in the control of mitotic progression and chromosome segregation. Expression of this protein has been related with a wide variety of human cancers [57]. The Nek2 kinase forms a complex with the PP1 holoenzyme. The protein is activated by autophosphorylation but is inactivated by constant PP1 dephosphorylation within the complex [58]. The PP1 inhibitor-2 (I-2) protein activates the kinase by binding to the Nek2::PP1 complex. In *in vitro* assays MC-LR binds to the complex leading to similar activation of Nek2 kinase [58]. Likewise this biochemical interaction can occur at the cellular level with implications in the viability of cells, tissue injury and tumor development.

The nuclear phosphoprotein P53 is induced in response to cellular stress. It plays a role as a transcriptional trans-activator in DNA repair, apoptosis and tumor suppression pathways [59]. The protein is a substrate of PP2A [60] and therefore its activity is likely to be regulated, in part, by MC-LR. Although the MC-LR mediated P53 regulation via PP2A has not been demonstrated the toxin leads to an increase in expression of p53 gene/protein in apoptotic HepG2 cells, cultured hepatocytes, rat liver tissues and FL cells [59,61,62]. P53 is a regulator of the expression of the anti- and pro-apoptotic genes including members of the Bcl-2 family such as Bcl-2 and Bax. These proteins are potential players in p53 induced cell apoptosis through the involvement in the outer-membrane-permeabilization transition (MPT) of the mitochondria, thereby triggering the process of apoptosis [63]. Significant increase in the expression of cell-cycle regulation and cellular proliferation genes were observed in the liver of homozygous p53 deficient knockout mice treated with MC-LR, in comparison to age-matched wild-type controls [64]. Moreover livers from the Trp53-deficient, MC-LR group displayed greater hyperplastic and dysplastic changes as well as increases in Ki-67 and phosphohistone H3 (mitotic marker) immunoreactivity. This work indicates that p53 has an important function in preventing the proliferative response and tumor promotion associated with chronic, sublethal MC-LR exposure [64]. It is probable that p53 is activated in cells exposed to the toxin, as a defense mechanism of cells to processes that lead to cellular proliferation and tumorigenesis, therefore triggering apoptosis (Figure 2).

Another protein putatively involved in MC toxicity is Bcl-2. Lin *et al*. [65] suggested that Bcl-2 is a direct substrate of PP2A and the anti-apoptotic activity of the protein is mediated by phosphorylation at the endoplasmic reticulum (ER). The phosphorylated form of the protein is degraded via proteasome in the ER leading to the sensitization of cells to several ER related stresses and apoptosis [65]. The protein is present in the ER and mitochondria and is likely to be regulated via PP2A or p53. Its involvement in cell apoptosis processes makes it a putative player in the mechanisms of MC toxicity (Figure 2).

Mitogen-activated protein kinases (MAPKs) regulate the expression of proto-oncogenes which on the other hand regulate the transcription of genes involved in the growth and differentiation [66]. Expression of MAPKs is mediated by PP2A [66] and are likely to be regulated by MC (Figure 2). MAPKs were induced in apoptotic HEK293-OATP1B3 cells exposed to MC-LR suggesting for a role of these proteins in cell apoptosis mediated by the toxin [67]. The expression of three proto-oncogenes c-fos, c-jun and c-myc were reported to increase in liver, kidney and testis of Wistar rats injected intravenously with MC-LR, with higher levels registered in liver [68]. Expression of these genes, pointed by the authors as a possible mechanism for the tumor-promoting activity of the toxin, could be controlled by MAPKs.

### 3.3. Oxidative Stress

A biochemical feature of MC toxicity is the production of reactive oxygen species (ROS). This ROS generation has been reported using *in vitro* systems such as human hepatoma cell line (HepG2) [69], fish cell lines RTG-2 and PLHC-1 [70], lymphocytes [13] and human erythrocytes [71], as well as in several *in vivo* studies in mice and rat liver, heart and reproductive system [72–76]. It is intrinsically related with mitochondria metabolism and can conduct to cell death by necrosis or apoptosis and to genotoxicity. Likewise it is believed to be involved in a series of pathologies such as impairment of the blood pumping-function of heart [75] and diseases in liver and kidney [73,74]. The underlying mechanisms of MC mediated ROS production and cell injury are however poorly characterized [74]. Ding *et al*. [72] first reported the surge of Ca^2+^ in the mitochondria of cultured rat hepatocytes, as the first event preceding apoptosis induced by MC-LR. The surge of calcium led to the onset of MPT with subsequent cell death. The onset of MPT represents an abrupt increase of permeability of the inner mitochondrial membrane to solutes with a molecular mass of less than 1,500 Da [77–79]. Following the onset of MPT, three important cellular events can occur (i) elevation of ROS formation, (ii) loss of mitochondrial membrane potential (MMP), and (iii) release of apoptotic factors from mitochondria, such as cytochrome *c*, triggering the execution of apoptosis [79]. Thereby the authors proposed that mitochondrial Ca^2+^ is one of the underlying mechanisms in MC-LR induced onset of MPT and cell death. Exposure of *Carassius auratus* lymphocytes *in vitro* to the variant MC-RR (10 nmol L^−1^) caused a massive Ca^2+^ influx, elevation of ROS and subsequent disruption of mitochondrial membrane potential and depletion of ATP [13]. The study supports the role of Ca^2+^ in MC activity and MC variants may have similar ways of action. Another plausible mechanism for ROS generation is the increase of NADPH oxidase activity. Nong *et al*. [69] verified the up-regulation of the CYP2E1 mRNA, an isoform of cytochrome P450 that exhibits NADPH oxidase activity, concomitant with the oxidative stress in HepG2 cells induced by MC-LR. Recently other molecules have been reported to be related with MC-LR mediated mitochondria dysfunction and oxidative stress. The pro-apoptotic proteins Bax and Bid were up-regulated in *in vivo* hepatocytes of mice liver upon oxidative stress induced by MC-LR. This up-regulation of the proteins was concomitant with the loss of mitochondrial membrane potential and cell apoptosis [73]. It is known that pro-apoptotic proteins associate to create pores in mitochondria membrane therefore being capable of inducing MPT [73]. Additionally to Ca^2+^ and CYP2E1 these proteins are potential players in MC-LR induced oxidative stress and cell apoptosis (Figure 2). Moreover it is well-established that oxidative stress can activate the c-Jun *N*-terminal protein kinase (JNK) pathway in the context of various stimuli or diseases [74]. The protein kinase may act through downstream substrates, such as AP-1 and Bcl-2 family molecules [74], to induce apoptosis, especially via the mitochondria-dependent apoptotic pathway. Likewise Wei *et al*. [74] verified that JNK activation affects some crucial enzymes of energy metabolism and leads to mitochondria dysfunction induced by MC-LR. Activity of JNK may, in this way, contribute significantly to hepatocyte apoptosis and oxidative liver injury by MC-LR. La-Salete *et al*. [80] demonstrated that MC-LR interacts directly with isolated mitochondria of rat kidney. The toxin led to a strong decrease of the transmembrane potential as a consequence of the inhibition of redox complexes [80]. The results point for a bioenergetic lesion in the mitochondria of kidney cells induced by MC-LR which can contribute significantly to the renal injury induced by the toxin. Part of the effects reported by the authors can be explained by the direct interaction of the toxin with the ATPase and aldehyde dehydrogenase of the mitochondria, as proposed by Mikhailov *et al*. [81] and Chen *et al*. [82], respectively. Due to the relevance of oxidative stress in MC toxicity, antioxidant compounds such as vitamin E and its analog trolox could be considered in prevention and therapy of animals exposed to the toxin. The compounds have been shown to quench free radicals, decrease the oxidative stress and reduce histological damage in the fish species tilapia exposed to MC [8,83].

### 3.4. Induction of Neutrophil-derived Chemokine

The excessive activity of leukocytes in lesion areas of the body has been regarded to induce tissue injury and microvascular dysfunction. This is the result of the release, by the neutrophils, of proteolytic enzymes and reactive oxygen and nitrogen metabolites which will promote tissue damage [84]. Similarly to the majority of the lesions that occurs in humans and animals, the MC toxicity is also characterized by the influx of neutrophils to affected organs [85–87]. Nevertheless, the role of neutrophils in the pathogenesis of MC has not been established. Recently Kujbika *et al*. [88,89] proposed that MC function as a chemotactic agent in neuthrophils playing therefore an important role in inflammatory responses. MC-LA, MC-YR and MC-LR induced the expression of the chemokines interleukin-8 (IL-8) and cytokine-induced neutrophil chemoattractant-2αβ (CINC-2αβ) as well as the extracellular ROS levels in *in vitro* human and rat neutrophils [89]. MC-LR increased the L-selectin and β2-integrin chemokines expression in mice leukocytes and all MC variants induced calcium influx with different kinetic profiles. Through the evaluation of the leukocyte-endothelial interactions in the presence of MC variants, the authors verified that only MC-LR could induce rolling and adherence of leukocytes, increase of L-selectin and β2-integrin expression but MC-LA, MC-YR and MC-LR induced migration of neutrophils and calcium influx with different kinetic profiles, therefore demonstrating the neutrophil chemotaxis and Ca^2+^ mobilization mediated by MC [88].

## 4. MC Biotransformation and Excretion

Kondo *et al*. [90] first demonstrated that MC could be synthetically converted into glutathione (GSH) and cystein conjugates. On examination of the 50% lethal dose (LD_50_) with intravenous injection using mice, both GSH and Cys conjugates showed reduction in toxicity compared with the native toxin. The authors further investigated the hepatic metabolism of MCs in mice and rats. MCs and several metabolites were identified in the livers by means of HPLC and Frit-FAB LC/MS. Among them, GSH and Cys conjugates of MCs were identified. Another metabolite was detected as a product of epoxidation followed by hydrolysis and sulfate conjugation in the Adda moiety and GSH conjugation in the Mdha moiety [90]. The conjugation of GSH with MCs was suggested to play a role in the metabolic pathway leading to detoxification of MCs [90]. Pflugmacher *et al*. [91] showed that MC-LR conjugated to glutathione is mediated by soluble glutathione S-transferase in various aquatic organisms ranging from plants (*Ceratophyllum demersum*), invertebrates (*Dreissena polymorpha*, *Daphnia magna*) up to fish eggs and fish (*Danio rerio*), stating that the pathway for xenobiotic metabolism expressed in all major groups of organisms is involved in the detoxication of MC. Zegura *et al*. [92] verified an increase of the levels of reduced GSH during the exposure of HepG2 cells to MC-LR, after an initial decay. Concomitant with this increase was the expression of glutamate-cysteine ligase (GCL), the rate-limiting enzyme of GSH synthesis indicating an increased rate of the de novo synthesis of GSH [92]. Normal or elevated levels of intracellular GSH were correlated with no additional DNA damage or the repair of existing damage. The work points out the importance of GSH in the amelioration of MC-LR genotoxicity which can be explained by its capacity to inhibit the activity of the toxin by forming MC-GSH conjugates or by its antioxidant activity. In the metabolism of xenobiotics the first reactions are catalyzed by cytochrome p450 oxidases and GSTs which respectively introduce reactive or polar groups into the xenobiotics and conjugate the modified toxins with glutathione. The modified toxins can be further excreted from cells and eliminated through the bile system or metabolized in cells prior to its elimination. GST has been the enzyme most studied regarding the biotransformation of MC. Nevertheless the underlying pathways and enzymes of MC biotransformation/excretion were not identified yet. In mammals, three major families of GSTs have been identified: the cytosolic GSTs (including seven classes, namely alpha, mu, pi, theta, sigma, omega, and zeta), the mitochondrial GST (kappa class) and microsomal GSTs [93]. A distinct GST isoform, which has no homologues in mammals, was found in fish and named as rho class in red sea bream (*Pagrus major*) [94]. Li *et al*. [95] studied the GST mRNA abundance in liver, kidney and intestine of Goldfish i.p. injected with microcystins extract at two doses (50 and 200 microgkg(-1)BW MC-LReq) by real-time PCR. The authors verified that the transcription of GST alpha isoform was suppressed in both liver and intestine, but induced in the kidney. The transcription of GST theta decreased in liver, kidney and intestine in the low-dose group and the transcription of GST pi isoform was suppressed in liver and intestine post-injection in both dose groups [95]. The work demonstrates that the transcription of GST isoforms varies in different ways within an organ and among organs of goldfish exposed to MCs. The relative changes of nine GST genes including cytosolic GSTs (rho, mu, theta, alpha and pi), mitochondrial GST (kappa) and microsomal GSTs (mGST1, mGST2 and mGST3) were studied after stimulation with MC-LR in the liver of the common carp *Cyprinus carpio* [96]. Increased levels of GST alpha, rho and mGST3 transcripts were detected at 6h post stimulation; the mu, theta and mGST2 transcripts were relatively stable; and all the GST transcripts except GST kappa and rho recovered to original levels compared with controls at 72 h. This work highlights the importance of the analysis of the different GSTs at different times post-stimulation for the identification of the isoforms putatively involved in MC biotransformation [96]. Wang *et al*. [97] studied the transcriptional response of liver genes related to phase I and phase II detoxification in the Nile tilapia (*Oreochromis niloticus*) fish exposed to MC-LR via intraperitoneal injection. The authors reported the increase in the soluble GST isoform (sGST) mRNA expression but in contrast, the levels of cytochrome P450 1 A (CYP1A) mRNA expression were unchanged with MC-LR treatment supporting the putative involvement of the GST and the importance of the phase II in the cyanotoxin detoxication through the conjugation pathway [97]. The authors also suggest that hepatocyte proteins coping with oxidative stress such as glutathione peroxidase and the uncoupling protein 2 have some auxiliary effect.

Multi-xenobiotic resistance (MXR) is a physiological process analogous to the multidrug resistance (MDR) reported in tumor cells. It is a defense mechanism that contributes to the decrease in intracellular concentrations of many unrelated but cytotoxic compounds. The processes are characterized by the increase in the expression of multidrug transporters such as the P-glycoprotein (P-gp). In mammalian tumor cells this increase of P-gp expression leads to a drop in the accumulation of chemotherapeutical drugs as well as the formation of resistant tumor cells [98]. P-gp is found endogenously in specialized epithelial tissues involved in secretion and excretion such as the mammalian gut, liver, and kidney, as well as on endothelial cells of capillary blood vessels at the blood-brain barrier [99]. P-gp acts as an energy-dependent pump to translocate a wide variety of structurally and functionally diverse substrates. These compounds tend to be moderately hydrophobic, planar, natural products which are often substrates or metabolites of the detoxification enzymes cytochrome P450 and GST [99]. The expression of the human P-gp homologous protein was found to increase in gills, liver and brain of the fish species *Jenynsia multidentata* after exposure to MC-LR, in a time and dose dependent manner [100]. The work indirectly associates P-gp expression with a defence mechanism to reduce the concentration of the toxin in the fish tissues [100]. The involvement of P-gp on the excretion of MC-LR in the freshwater mussel *Dreissena polymorpha* was evidenced through the analysis of P-gp gene expression and the analysis of the efflux and accumulation version of the compound rhodamine [101]. P-gp expression was enhanced after 1 h exposure but no changes were detected after longer (72 h) exposure. Nevertheless P-gp enzyme activity showed a significant increase with exposure time. The concomitant decrease of the concentration of the toxin in the tissues of animals during the 72 h exposure, after a peak of accumulation in the first hour of exposure indicates that the protein is putatively involved in the excretion of the toxin.

## 5. Conclusions

MC are potent hepatotoxins, with genotoxic properties. They are strong inhibitors of the serine/threonine phosphatases PP1 and PP2A. This is probably the main mechanism of action of these toxins through which alters cell’s metabolism and triggers a cascade of events leading to the necrosis or apoptosis of animal cells (Figure 2). MC regulate the activity of protein kinases by direct PP1 and PP2A inhibition. This can have a strong impact in the activity of phosphoproteins, DNA repair systems and gene expression. On the other hand the cyclic peptide seems to interact with the mitochondria of animal cells triggering the oxidative stress and apoptosis. Moreover the molecule can be inactivated in the cells in a process that implicates the conjugation with glutathione (Figure 2). MC toxicity is a multi-pathway process and regardless the recent achievements the molecular mechanisms underlying MC toxicity remain elusive and a lack of knowledge persists regarding the specific MC target/interacting proteins, the signaling pathways triggering the cell’s response and the downstream pathways of toxicity and cell’s injury. In this way complementary genomics and proteomics are promising approaches to achieve a comprehensive characterization of the MC toxicity in animal cells.

## Figures and Tables

**Table 1.** List of proteins expressed in animal cells with functions in the processes of MC up-take, toxicity and biotransformation.

				**MC toxicity**	

**Protein**	**Activity**	**Biological function**	**Presence of MC**	**Experimental approach**	**References**
PP1, PP2A	serine/threonine protein phosphatase	regulation of protein activity	inhibition of activity	MC-PP interactions	[11,42]
DNA-PK	serine/threonine protein kinase	DNA repair	decrease activity	activity of DNA repair synthesis from cell extracts and purified DNA-PK	[49,51]
CaMKII	serine/threonine protein kinase	cell signalling	increase in activity	exposure of primary hepatocytes to MC	[56]
NeK2	serine/threonine protein kinase	cell signaling	increase in activity	activity of purified NeK2:PP1 complex	[58]
P53	transcription factor	cell cycle, tumor supression	increase of protein/gene expression	exposure of HepG2, FL cell lines, primary hepatocytes and *in vivo* liver tissues to MC	[59,61,62]
Bcl-2	regulation of mitochondrial apoptosis-induced channel (MAC)	mitochondrial outer membrane permeabilization, apoptosis	decrease of protein/gene expression	exposure of primary hepatocytes, *in vivo* liver tissues and FL cells to MC	[59,61]
MAPKs	serine/threonine protein kinase	signal transduction, cell proliferation and differentiation	increase of gene expression	exposure of HEK293-OATP1B3 cell line to MC	[67]
NADPH oxidase	electron transfer to superoxide	production of ROS	increase of gene expression	exposure of HepG2 cell line to MC	[69]
Bax, Bid	regulation of mitochondrial apoptosis-induced channel (MAC)	mitochondrial outer membrane permeabilization, apoptosis	increase of protein expression	*in vivo* exposure of liver tissues to MC	[73]
JNK	MAPK	signal transduction, cell proliferation and differentiation	increase in protein expression	*in vivo* exposure of liver tissues to MC	[74]
IL-8, CINC-2αβ, L-selectin, β2-integrin	chemotactic cytokines	chemotaxis, inflammatory reactions	increase in protein/gene expression	*in vitro* exposure of neutrohils to MC	[88,89]
OATP	plasma membrane transporter	transport of organic anions	nd (a)	exposure of transfected *Xenopus laevis* oocytes and Oatp1b2-null mice to MC	[36,38]
GST	gluthatione S-transferase	metabolism of endogenous compounds and xenobiotics	differential expression of GST genes	*in vivo* exposure of fish liver tissues to MC	[96,97]
P-glycoprotein	plasma membrane transporter	cellular excretion of cytotoxic compounds	increase of gene expression and protein activity	*in vivo* exposure of fish liver, gills and brain tissues; fresh water mussel to MC	[100,101]

(a) Not determined.
